# A Multicenter Epidemiological Study on Second Malignancy in Non-Syndromic Pheochromocytoma/Paraganglioma Patients in Italy

**DOI:** 10.3390/cancers13225831

**Published:** 2021-11-20

**Authors:** Letizia Canu, Soraya Puglisi, Paola Berchialla, Giuseppina De Filpo, Francesca Brignardello, Francesca Schiavi, Alfonso Massimiliano Ferrara, Stefania Zovato, Michaela Luconi, Anna Pia, Marialuisa Appetecchia, Emanuela Arvat, Claudio Letizia, Mauro Maccario, Mirko Parasiliti-Caprino, Barbara Altieri, Antongiulio Faggiano, Roberta Modica, Valentina Morelli, Maura Arosio, Uberta Verga, Micaela Pellegrino, Luigi Petramala, Antonio Concistrè, Paola Razzore, Tonino Ercolino, Elena Rapizzi, Mario Maggi, Antonio Stigliano, Jacopo Burrello, Massimo Terzolo, Giuseppe Opocher, Massimo Mannelli, Giuseppe Reimondo

**Affiliations:** 1 Department of Experimental and Clinical Biomedical Sciences “Mario Serio”, University of Florence, 50139 Florence, Italy; letizia.canu@unifi.it (L.C.); giuseppina.defilpo@unifi.it (G.D.F.); mario.maggi@unifi.it (M.M.); massimo.mannelli@unifi.it (M.M.); 2 Centro di Ricerca e Innovazione sulle Patologie Surrenaliche, AOU Careggi, 50134 Florence, Italy; tonino.ercolino@unifi.it (T.E.); elena.rapizzi@unifi.it (E.R.); 3 Internal Medicine, Department of Clinical and Biological Sciences, San Luigi Gonzaga Hospital, University of Turin, Orbassano, 10043 Turin, Italy; soraya.puglisi@unito.it (S.P.); m.luconi@unifi.it (F.B.); a.pia@sanluigi.piemonte.it (A.P.); massimo.terzolo@unito.it (M.T.); giuseppe.reimondo@unito.it (G.R.); 4 Statistical Unit, Department of Clinical and Biological Sciences, University of Turin, Orbassano, 10143 Turin, Italy; paola.berchialla@unito.it; 5 Familial Cancer Clinic, Veneto Institute of Oncology IOV – IRCCS, 35128 Padua, Italy; francesca.schiavi@iov.veneto.it (F.S.); massimiliano.ferrara@iov.veneto.it (A.M.F.); stefania.zovato@iov.veneto.it (S.Z.); 6 Oncological Endocrinology Unit, IRCCS-Regina Elena National Cancer Institute, 00128 Rome, Italy; marialuisa.appetecchia@ifo.gov.it; 7 Oncological Endocrinology Unit, Department of Medical Sciences, University of Turin, 10126 Turin, Italy; emanuela.arvat@unito.it; 8 Secondary Arterial Hypertension Unit, Department of Translational and Precision Medicine, Sapienza University of Rome, 00161 Rome, Italy; claudio.letizia@uniroma1.it (C.L.); luigi.petramala@uniroma1.it (L.P.); antonio.concistre@uniroma1.it (A.C.); 9 Endocrinology, Diabetology, and Metabolism Unit, Department of Medical Sciences, University of Turin, 10126 Turin, Italy; mauro.maccario@unito.it (M.M.); mirko.parasiliticaprino@gmail.com (M.P.-C.); 10 Division of Endocrinology and Diabetes, Department of Internal Medicine I, University Hospital, University of Würzburg, 97080 Würzburg, Germany; Altieri_B@ukw.de; 11 Endocrinology, Department of Clinical and Molecular Medicine, Sant’Andrea Hospital, Sapienza University of Rome, 00189 Rome, Italy; antongiulio.faggiano@uniroma1.it (A.F.); antonio.stigliano@uniroma1.it (A.S.); 12 Division of Endocrinology, Department of Clinical Medicine and Surgery, ENETS Center of Excellence, University “Federico II” of Naples, 80138 Naples, Italy; robertamodica@libero.it; 13 Endocrinology Unit, Fondazione IRCCS Ca' Granda Ospedale Maggiore Policlinico, Department of Clinical Sciences and Community Health, University of Milan, 20122 Milan, Italy; morellivale@yahoo.it (V.M.); maura.arosio@unimi.it (M.A.); uverga@gmail.com (U.V.); 14 Division of Endocrinology, Diabetology and Metabolism, Santa Croce and Carle Hospital, 12100 Cuneo, Italy; micaela13.pellegrino@gmail.com; 15 Endocrinology, Diabetology and Metabolism Diseases Unit, AO Ordine Mauriziano, 10128 Turin, Italy; prazzore@mauriziano.it; 16 Endocrinology Unit, AOU Careggi, 50134 Florence, Italy; 17 Department of Experimental and Clinical Medicine, University of Florence, 50139 Florence, Italy; 18 Division of Internal Medicine and Hypertension Unit, Department of Medical Sciences, University of Turin, 10126 Turin, Italy; jacopo.burrello@unito.it; 19 Veneto Institute of Oncology, IRCCS, 35128 Padua, Italy; giuseppe.opocher@gmail.com

**Keywords:** pheochromocytoma, paraganglioma, epidemiology, genetic analysis, mortality, surveillance

## Abstract

**Simple Summary:**

As no previous studies had assessed the risk of second malignant tumors in patients with pheochromocytomas/paragangliomas (PPGLs), we aimed to evaluate whether these patients could have an increased risk of additional malignancy, comparing them with patients in the general population who had a first malignancy and developed a second malignant tumor. We demonstrated that PPGL patients had higher incidence of additional malignant tumors and the risk of developing a second malignant tumor increased with age at diagnosis. As the main tumors were prostate, colorectal and lung/bronchial cancers in males, and breast cancer, differentiated thyroid cancer and melanoma in females, our findings could have an impact on the surveillance strategy.

**Abstract:**

No studies have carried out an extensive analysis of the possible association between non-syndromic pheochromocytomas and paragangliomas (PPGLs) and other malignancies. To assess >the risk of additional malignancy in PPGL, we retrospectively evaluated 741 patients with PPGLs followed-up in twelve referral centers in Italy. Incidence of second malignant tumors was compared between this cohort and Italian patients with two subsequent malignancies. Among our patients, 95 (12.8%) developed a second malignant tumor, which were mainly prostate, colorectal and lung/bronchial cancers in males, breast cancer, differentiated thyroid cancer and melanoma in females. The standardized incidence ratio was 9.59 (95% CI 5.46–15.71) in males and 13.21 (95% CI 7.52–21.63) in females. At multivariable analysis, the risk of developing a second malignant tumor increased with age at diagnosis (HR 2.50, 95% CI 1.15–5.44, *p* = 0.021 for 50–59 vs. <50-year category; HR 3.46, 95% CI 1.67–7.15, *p* < 0.001 for >60- vs. <50-year). In patients with available genetic evaluation, a positive genetic test was inversely associated with the risk of developing a second tumor (HR 0.25, 95% CI 0.10–0.63, *p* = 0.003). In conclusion, PPGLs patients have higher incidence of additional malignant tumors compared to the general population who had a first malignancy, which could have an impact on the surveillance strategy.

## 1. Introduction

Pheochromocytomas and paragangliomas (PPGLs) are rare tumors arising from the neural crest [1]. Pheochromocytomas (PCCs) and thorax/abdominal paragangliomas (PGLs) derive from sympathetic ganglia, whereas head and neck PGLs (HNPGLs) derive from parasympathetic ones [2].

Up to 70% of PPGLs are caused by germline or somatic genetic variants in one of the susceptibility genes [3]. Depending on the transcription profile, PPGLs are divided into two main clusters: cluster 1 includes genes involved in pseudohypoxia signaling (*SDHA, SDHB, SDHC, SDHD, SDHAF2, VHL, FH, EPAS1*), and cluster 2 includes genes related to the activation of kinase signaling (*NF1, RET, TMEM127, MAX, HRAS*) [3,4].

Until a few years ago, the association of PPGL with other solid tumors was reported only in neurofibromatosis type 1 (NF1), multiple endocrine neoplasia type 2 (MEN2) and von Hippel Lindau (VHL) syndrome. However, non-chromaffin tumors have recently been reported in patients with PPGL without any of these syndromic diseases. In fact, *SDHx* mutations have been associated with renal cell carcinomas (RCCs) [5], gastrointestinal stromal tumors (GISTs) [6,7] and pituitary adenomas (PAs) [6]. *SDHx* mutated RCCs represent less than 0.5% of all renal carcinomas [8], whereas 30% of GISTs are associated with *SDHA* mutations [9].

The presence of *SDHC* promoter hypermethylation has also been observed in patients affected by SDH-deficient GIST without somatic *SDHx* mutations [10]. *MAX* mutated patients are rarely affected by pituitary adenomas [11] and RCC has been reported in *TMEM127* [12] and *FH* [13] mutated patients. The prevalence of *SDHx* mutations in pituitary adenomas is very low (0.3–1.8%) [14] and the majority are functional macroadenomas [15].

The data on the association between non-chromaffin tumors and PPGLs with or without mutations in any of the PPGL susceptibility genes are heterogeneous. A great deal of interest has been placed on the association with *SDHx* mutations, and these tumors have been defined as *SDH*-deficient tumors [9]. Some studies have reported an association between PPGLs and other solid tumors in non-genotyped patients. On the other hand, other studies have reported the presence of GISTs, RCCs or pituitary adenomas in *SDH*-mutated patients, but without proving a causal relationship between the *SDHx* mutation and tumor occurrence.

We searched the current literature for studies (Supplemental Table S1 [16]) on patients affected by PPGL and/or other tumors, including patients who were carriers or not of mutations in any of the PPGL susceptibility genes. Any genetic alteration should be clear from the immunohistochemistry (IHC) and/or loss of heterogeneity (LOH) in tumor tissue. We found that IHC and LOH on tumor tissue revealed a mutation in 9.6% (784/8159) and 34% (143/420) of cases, respectively. IHC was more widely used, but LOH more frequently identified non-chromaffin tumors due to mutations in susceptibility genes.

The aims of this retrospective, multicentric study were to assess whether patients with PPGLs have an increased risk of additional malignant tumors compared with the general population, and to identify the predisposing factors.

## 2. Materials and Methods

### 2.1. Subjects

We evaluated the prevalence and incidence of an additional malignant tumor in 741 patients affected by PPGLs followed-up in 12 referral centers in Italy, listed in Appendix B. Patients with confirmed biochemical and/or histopathological diagnosis of PPGL were included, while those presenting with known hereditary syndromes, such as VHL, MEN2 and NF1, were excluded. The median duration of follow-up was 48 months (12–108).

Genetic analysis was considered as assessed if at least *SDHx*, *MAX* and *TMEM127* genes were analyzed. Data on patients diagnosed between 1990 and 2019 were collected retrospectively by local investigators in a computerized database. Most patients were diagnosed between 2009 and 2019 (46.8%). All patients gave their informed consent to the collection of data according to the local ethics committee indications (Registry and Repository of biological samples of the European Network for the Study of Adrenal Tumors (ENS@T).

We collected the following data: demographics, date of diagnosis, metanephrine (MN), normetanephrine (NMN) and methoxytyramine (MTX) levels, detection of malignant tumors before, after or within the same year of the PPGL diagnosis, family history of tumors, smoking (yes/no answers), drinking (female >1 alcholic unit (A.U.)/per day, male >2 A.U./per day) and toxic exposure (yes/no answers). Toxic exposure was classified as occupational exposure to toxic substances such as pesticides, polychlorinated biphenyls, asbestos, radon and lead-based paint.

The incidence of a second malignant tumor found in our series was compared to that of the general Italian population (data from Italian Network of Cancer Registries—AIRTUM registry 2019) [17]. Age was reported as a categorical variable in line with what is reported in the AIRTUM registry. The comparison was carried out considering the associated malignant tumors as a second event, taking into account that the 2017 World Health Organization (WHO) classification includes PPGLs among malignant tumors [18,19,20].

### 2.2. Statistical Analysis

Continuous variables were presented using the median and the interquartile range (IQR) as measure of variability; categorical variables were presented with frequencies and percentages. Differences between groups were analyzed with the Mann –Whitney test for continuous variables and the chi-squared test, or Fisher test when appropriate, for categorical variables. To evaluate the factors associated with the risk of second malignancy after the diagnosis of the chromaffin pathology, a univariable analysis was carried out to estimate the hazard ratio (HR) and the corresponding 95% confidence interval (95% CI) with the Cox proportional hazard model. In the Cox proportional hazard models, age was entered as a categorical variable. A final multivariable model was developed based on clinical discussion and statistical selection procedures. Model selection was performed using an automatic approach based on the Akaike Information Criteria (AIC) method [21]. Given the large number of covariates, a genetic algorithm was used to explore the candidate set of models. Model goodness of fit was computed with reference to the Brier score (the closer to 0, the better) and the Somers’ Dxy Index, which assesses the predictive discrimination derived from the set of predictor variables included in the model. To compute the Somers’ Dxy index, the predictive survival time was used. To account for the degree of optimism in model accuracy evaluations induced by the use of the same data source for training and testing purposes, all goodness of fit indexes were computed using a bootstrap procedure (1000 runs). The Schoenfeld residual-based method was used to verify the assumption of proportionality of the risks. The significance level was set at *p* < 0.05. Incidence of second malignant tumors in the study sample was compared with the incidence in Italian patients who had a first malignancy and developed a second malignant tumor. The standardized incidence ratio (SIR) was computed, which is the ratio of the observed number of second malignancies in the study sample to the number of the cases expected according to a set of reference incidence rates. The number of expected tumors was computed by multiplying the number of person-years in the cohort by the national cancer incidence rates, specified for sex and 5-year-age-group and calendar year. Incidence rates by sex and age and calendar year of second malignant tumor of the Italian population were obtained from the AIRTUM database [17]. An SIR greater than 1 means a higher incidence than expected in the reference population. Finally, exact Poisson 95% CIs were computed. Data were analyzed with R version 3.5.0.

## 3. Results

This study included 741 PPGL patients, of whom 415 (56.0%) were female, with a median age at diagnosis of 49 years (36–60).

Patients’ characteristics are reported in Table 1.

Genetic analysis was performed in 69.5% of patients and 32.2% were mutation carriers: 16.7% *SDHD*, 8.7% *SDHB*, 2.3% *MAX*, 2.1% *TMEM127*, 1.4% *SDHC*, 0.8% *SDHA* and 0.2% *SDHAF2*. A total of 26.6% of the patients belonged to cluster 1, and 4.5% to cluster 2. Patients’ characteristics are reported in Table 2.

**Figure 1:**
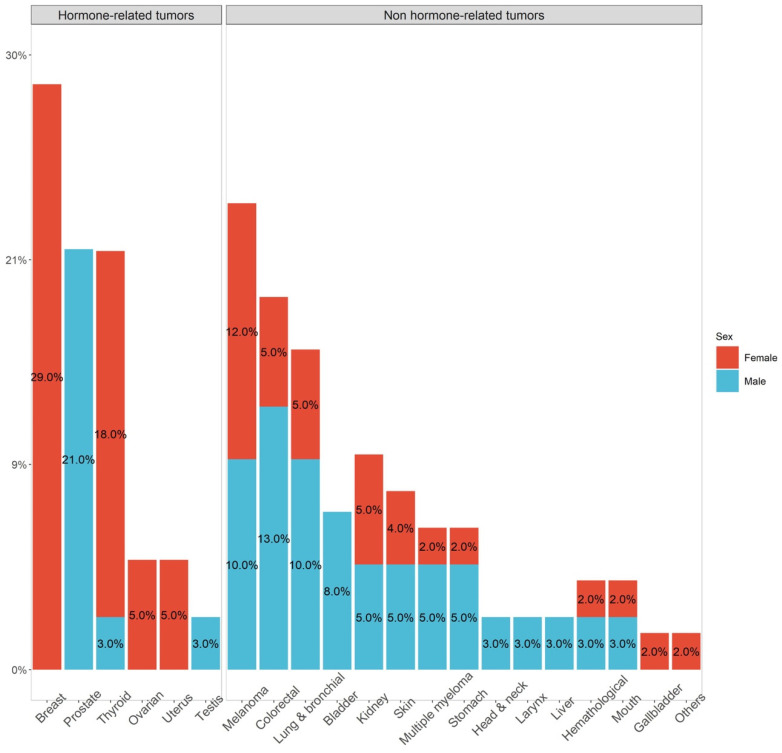
Frequency of second malignant tumor, according to gender (in red females, in blue males) divide into hormone-related and non-hormone-related tumors.

Ninety-five (12.8%) patients developed a second malignant tumor: mainly breast cancer, differentiated thyroid cancer (DTC) and melanoma in females and prostate cancer, colorectal cancer and lung and bronchial cancer in males (Figure 1).

Twenty-nine (30.5%) of second malignant tumors were discovered after the diagnosis of PPGLs. Comparing our series with the general population [17], the standardized incidence ratio (SIR) of the whole series was 9.59 (95% CI 5.46–15.71) in males, and 13.21 (95% CI 7.52–21.63) in females. The same figure was also observed in the group of subjects who were genetically tested: 7.86 (95% CI 3.44–15.56) in males and 15.71 (95% CI 8.26–27.21) in females.

Only 18% of patients who developed a second malignancy carried a germ-line mutation, which was present in 34% of individuals without a second malignant tumor (*p* = 0.01). Comparing the 646 patients without second malignant tumors with the 95 patients who developed a second malignant tumor (Table 3), the latter patients were more frequently older (*p* < 0.001), had less frequently germline mutations (*p* = 0.01), with a minor frequency in genes involved in pseudohypoxia signaling (11.1% of patients with second malignant tumors belonging to cluster 1 vs. 29.8% of patients without second malignant tumors, *p* = 0.006). No significant difference was found considering the urinary metanephrine and normetanephrine levels comparing patients with and without second malignant tumors (*p* 0.873 and *p* 0.522, respectively).

The risk factors associated with the development of second malignant tumors after the diagnosis of PPGLs were assessed by univariable analysis (Table 4). The analysis revealed an association with age (HR 2.27, 95% CI 1.13–4.53, *p* = 0.021 for the 50–59 age category vs. <50 age category; HR 2.22, 95% CI 1.05–4.69, *p* = 0.036 for the over 60 vs. <50 age category).

In the univariable analysis, germline mutations were associated with a lower risk of developing a second malignant tumor (HR 0.27, 95% CI 0.11–0.63, *p* = 0.003). The presence of mutations occurring in the susceptibility genes belonging to cluster 1 (HR 0.31, 95% CI 0.13–0.73, *p* = 0.008), but not to cluster 2, was also inversely associated with the risk of second tumors. Positive family history of cancer was associated with an increased risk of a second malignant tumor (HR 1.80, 95% CI 1.03–3.14, *p* = 0.04).

In the multivariable analysis, the risk of developing a second malignant tumor increased with age at diagnosis (HR 2.50, 95% CI 11.5–5.44, *p* = 0.021 for 50–59 vs. <50; HR = 3.46, 95% CI 1.67–7.15, *p* < 0.001 for the over 60 vs. <50) (Table 5A). In the series of patients with an available genetic evaluation, the association between age and risk of second tumor weakened, whereas a positive genetic test was strongly protective against developing a second tumor (HR 0.25, 95% CI 0.10–0.63, *p* = 0.003) (Table 5B).

A median of 6 (2–14) years elapsed between the diagnosis of PPGLs and the appearance of a second malignant tumor with a progressive reduction in the risk of developing a second tumor of 7% per year (HR 0.94, 95% CI 0.90–0.97, *p* < 0.001).

## 4. Discussion

In this study, we observed a higher risk of developing second malignant tumors in patients with PPGLs compared with the general population in Italy. The risk was greater in patients affected by sporadic PPGLs compared to genetically driven PPGLs. The presence of a known mutation in any of the susceptibility genes for PPGLs was actually a protective factor against developing a second malignant tumor.

The analysis revealed a higher incidence of second malignancies in our series, both in males and females, with an approximately 9 and 13 times higher risk, respectively, confirming previous preliminary findings in a small sample (110 PCC and 11 PGL) with sporadic and familial tumors [22]. The risk appears higher than expected since we compared the incidence of second malignant tumors in our population with that in the general population who had a first malignancy and developed a second malignant tumor. The comparison was conducted in view of the new WHO classification which includes all PPGLs among malignant tumors [18].

We focused on second malignant tumors both due to the greater clinical interest of these tumors compared to benign ones, together with the availability of incidence data on only malignant tumors in the general population [17]. The most frequently reported association in the literature concerns GIST, RCC and pituitary adenoma. The data reported in the literature are rather heterogenous, with studies conducted on patients suffering from non-chromaffin tumors with a negative history of PPGLs and lacking a genetic analysis for known susceptibility genes but with the tumor tissue analysis of *SDHx* mutations. Other authors have described the appearance of non-chromaffin tumors in patients with previously sporadic or familial PPGLs. Moreover, in some studies the association between PPGL and second tumors or between *SDHx* mutations and non-chromaffin tumors, was not supported by immunohistochemical analysis or tissue gene sequencing. In addition, the interpretation of the immunohistochemical analysis for SDHB and/or SDHA on PPGL tissues is not always univocal [23], as also happens in the tissues of other tumors. In our case series there was one GIST, one GH- secreting pituitary adenoma and five kidney lesions.

In the whole series, in females we found that the most frequent cancers associated with PPGLs were breast cancer, DTC and melanoma. In males, the most frequent tumors were prostate cancer, colorectal cancer and lung and bronchial cancer. In the general population, colorectal cancer is the second most frequent tumor (13%) after breast cancer (14%), followed by prostate, lung and bronchial cancer (all 11%) [17]. The high frequency of breast cancer is in line with findings observed in the general population, since it represents the most frequent neoplasia in the female population (30%), while DTC is the fourth (5%) [17]. The high incidence of DTC in our population may result from a selection bias of patients who were followed up in endocrinology care units, where thyroid evaluation is routinely performed. Melanoma, representing the third most frequent second neoplasia in our cohort, was in the youngest population (<50 years), the second most frequent in males (9%), immediately after testicular cancer (12%), whereas it was the third in females (7%), after breast cancer (40%) and DTC (16%) [17]. Interestingly, in our series almost 50% of patients with melanoma were older than 60. Melanoma has already been identified as one of the most frequent cancers associated with PPGLs in women in a study including 121 patients with PPGLs [22]. The association between PPGL and melanoma is interesting due to their common embryonic origin from the neural crest. The microphthalmia-associated transcription factor (MITF) is a transcription factor involved in the regulation of survival, proliferation and differentiation of the neural crest cells such as melanocytes [24]. Two studies [25,26] identified a germline variant of MITF, *p*.E318K, associated with an increased risk of melanoma and RCC. Castro-Vega et al. hypothesized that this variant might also contribute to the development of PPGL, which they found in 7 out of 555 patients with PPGL [27]. The breast cancer associated protein 1 (BAP1) gene is a tumor suppressor gene involved in cell cycle regulation, cell differentiation, cell death and DNA damage response [28]. Loss of BAP1 expression has been demonstrated in many other tumors including melanoma, mesothelioma and RCC. Maffeis et al. analyzed tissues of 56 PPGLs, demonstrating the loss of BAP1 expression also in PPGLs (2/22 PGL and 12/34 PCC) [29]. Only in a few cases has an association between DTC and sporadic/genetically inherited PPGL been described. To date, the relationship between DTC and PPGL remains to be clarified and is likely affected by a heterogeneous genetic background [30]. Currently only one case has been reported of prostate cancer SDHB negative at immunohistochemistry [31], while the association between prostate cancer and PPGLs has not been described. Interestingly, in our population one patient developed prostate cancer at 30 years old after a diagnosis of chromaffin disease.

Advanced age at diagnosis of PPGLs is a predisposing factor for the development of second malignant tumors, similarly to findings in the general population. However, a progressive 7% reduction per year in the risk of developing a second tumor has been observed with increasing time after a diagnosis of PPGL. We cannot exclude that the accurate diagnostic evaluation, starting from the initial diagnosis of chromaffin pathology, might facilitate the detection of unknown co-morbidities, including tumors in the early years of follow-up. Current data indicate a lifetime follow up in patients with PPGL familial forms and a 10-year follow up in patients with PPGL sporadic forms [32] which is also suitable for identifying incidentally detected second malignant tumors.

An intriguing result emerging from our analysis of the series is the role of the genetic profile. In the last decade, there has been growing interest in other tumors in patients with PPGLs. Most studies have evaluated the association between the second tumors and *SDHx* mutations. In line with the literature data, 30% of our patients were carriers of a germ-line mutation for PPGLs [5]. In view of the data on the association between *SDHx* and other tumors [33], we expected that second malignant tumors would be more frequent in patients with genetic forms of PPGLs, particularly belonging to cluster 1 with mutations of the *SDHx* genes. However, our analysis revealed that almost 82% of patients with second malignant tumors were affected by sporadic forms. This data could be explained by mutations in not yet identified PPGL susceptibility genes. Single nucleotide polymorphisms (SNPs) possibly play a protective role. SNPs are single nucleotide variations present in more than 1% of the population [34]. In Wilms’ tumor, in squamous cell carcinoma of the head and neck [35], and in breast cancer [36], SNPs in genes appear to be involved in the base excision repair (BER) complex, which is the main DNA repair mechanism in damage induced by reactive oxygen species (ROS) [37]. The protective role of SNPs is thus highly selective for a specific type of tumor; in fact, SNPs that reduce the risk of developing a type of tumor, conversely may play a promoting action for other tumor histotypes [35]. Similarly, mutations in the susceptibility genes for PPGLs might predispose the development of chromaffin diseases, while reducing the risk of other malignant tumors.

Another unexpected finding is that patients affected by a second malignant tumor less frequently belonged to cluster 1, which in our series mainly included *SDHx* genes. *SDHx* mutated cells present an impaired mitochondrial electron transport chain with increased ROS production. Accordingly, SDHx mutated cells shift to aerobic glycolysis (Warburg effect) [38]. This also happens in non-tumor cells bearing *SDHx* mutations which were forced towards glycolysis to maintain low levels of ROS, resulting in a less oxidative mutational environment that could protect against the development of non-chromaffin malignancies. This might justify why patients from cluster 1 developed a second malignant tumor less frequently than in cluster 2.

Secondly, in our series, no patient affected only by non-secreting parasympathetic lesion (HNPGL) developed a second malignant tumor. This result is in line with previous studies showing the role of catecholamines in tumorigenesis [39,40]. Interestingly, this data could also explain why patients belonging to cluster 1 are less affected by second malignant tumors. In fact, patients with parasympathetic lesions belong to cluster 1 and are not present in cluster 2. Despite this, in our study no significant difference was found between urinary metanephrine and normetanephrine levels comparing patients with and without second malignant neoplasm, probably due to the limited number of events.

Despite the associations described in the literature, to date, there is no indication to check for the presence of second tumors in patients affected by PPGLs and/or carriers of mutations in one of the susceptibility genes. Of the three most frequently associated tumors reported in the literature, RCC is the only one that can be found during the routine follow-up of our patients. Highlighting the presence of kidney lesions with an abdomen ultrasound is straightforward, while to identify pituitary adenoma, a dedicated contrasted-MRI is necessary. However, these lesions are generally larger than one centimeter, and in most cases, secreting. These characteristics could lead to the discovery of the lesion despite the lack of dedicated investigations during the follow-up. In order to rule out the presence of a GIST, an abdominal CT scan with contrast medium would be necessary, which is not usually done in a routine follow up.

Our findings suggest some modifications could be made to improve the follow-up procedures in females: (a) for breast cancer, a surveillance program for women between 50 and 69 years-old, which includes a mammogram every two years; (b) for DTC and melanoma, a neck ultrasound and a dermatological examination would be sufficient. In males: (a) annual detection of prostate specific antigen (PSA) value might be suggested; (b) for colorectal cancer, fecal immunochemical testing every two years for men between 50 and 75 years old.

Our study has some limitations. Due to the retrospective nature of the study not all missing data could be recovered. Anamnestic data were not collected in a standardized manner and not all patients underwent genetic analysis. Furthermore, there are also methodological differences in the genetic tests performed: traditional Sanger sequencing vs. new next-generation sequencing methods. We did not make tissue analysis of associated tumors to assess whether the germline mutation was responsible for the appearance of a second non-chromaffin neoplasia. Finally, the duration of the median follow-up (48, 12–108, months) was limited.

## 5. Conclusions

We believe that our study represents the most extensive evaluation of the prevalence of second malignant tumors in patients with PPGLs. Our main finding was that there is a higher incidence of second malignancies in patients affected by PPGLs compared to the general population.

Appropriate changes in the follow-up of patients with sporadic chromaffin tumors should thus be fostered, in order to identify a second tumor early. Finally, our results suggest the need for further efforts to identify new PPGL susceptibility genes.

## Figures and Tables

**Table 1:** Patient characteristics.

Characteristics	*n*. of Evaluated Patients	*N*
Sex Female Male	741	415/741 (56.0%) 326/741 (44.0%)
Age (years) at PPGL diagnosis, median	741	49 [IQR: 36–60]
Metastatic PPGL	612	54 (8.8%)
Functioning PPGL	572	379 (66.3%)
PPGL localization	741	
Abdominal PGL		172 (23.1%)
Mediastinal PGL		2 (0.3%)
HNPGL		3 (0.4%)
PCC		37 (5.0%)
Abdominal PGL + PCC		58 (7.8%)
Mediastinal PGL + HNPGL		56 (7.6%)
Abdominal PGL + HNPGL		5 (0.7%)
PCC + HNPGL		408 (55.1%)
Family history of tumor	727	264 (36.3%)
Risk factors		
Smoke	672	159 (23.7%)
Alcohol	678	32 (4.7%)
Exposure to toxic substances	625	29 (4.6%)
Genetic analysis	515	
Wild type		349 (67.8%)
*SDHD*		86 (16.7%)
*SDHB*		45 (8.7%)
*MAX*		12 (2.3%)
*TMEM127*		11 (2.1%)
*SDHC*		7 (1.4%)
*SDHA*		4 (0.8%)
*SDHAF2*		1 (0.2%)
Cluster 1	515	141 (26.6%)
Cluster 2	515	23 (4.5%)
Second malignant tumor	741	95 (12.8%)
Death		26 (3.5%)
Death for PCC/PGL		11 (1.5%)
Follow up months, median		48 [IQR: 12–108]

PPGL = pheochromocytoma and paraganglioma; IQR = interquartile range; PGL = paraganglioma; HNPGL = Head and neck paraganglioma; PCC = pheochromocytoma.

**Table 2:** Patient characteristics stratified by genetic mutation.

Characteristics	Patients with Mutation (*n*. 166)	Patients without Mutation (*n*. 349)	*p* Value
Sex			0.537
Female	92/166 (55.4%)	205/349 (58.7%)	
Male	74/166 (44.6%)	144/349 (41.3%)	
Age (years) at PPGL diagnosis, median	37 (IQR: 28–46.5)	52 (IQR: 41–61)	**<0.001**
Age (years) at second malignancy	57 (IQR: 47–65.5)	56.5 (IQR: 37.8–64)	0.527
Metastatic PPGL	21 (14.5%)	21 (6.8%)	**0.014**
Functioning PPGL	39 (30.5%)	202 (70.1%)	**<0.001**
HNPGL	92 (55.4%)	84 (24.1%)	**<0.001**
Family history of tumor	62 (38.5%)	174 (50.0%)	**0.020**
Risk factor: smoke	30 (20.5%)	90 (28.1%)	0.105
Risk factor: alcohol	4 (2.7%)	12 (3.7%)	0.781
Risk factor: exposure to toxic substances	0 (0.0%)	15 (4.8%)	**0.025**
Second malignant tumor			0.113
Before	4 (22.2%)	34 (49.3%)	
After	10 (55.6%)	23 (33.3%)	
Simultaneously	4 (22.2%)	12 (17.4%)	

PPGL = pheochromocytoma and paraganglioma; IQR = interquartile range; PGL = paraganglioma; HNPGL = Head and neck paraganglioma; PCC = pheochromocytoma. Statistically significant *p* are indicated in bold.

**Table 3:** Patient characteristics stratified by a second malignant tumor.

Characteristics	Patients with Second Malignant Tumors (*n* 95)	Patients without Second Malignant Tumors (*n* 646)	*p* Value
Sex Female Male	56/95 (58.9%) 39/95 (41.1%)	359/646 (55.6%) 287/646 (44.4%)	0.61
Age (years) at PPGL diagnosis, median	58 (IQR: 50–65.8)	47 (IQR: 35–58)	**<0.001**
Metastatic PPGL	5/76 (6.6%)	49/536 (9.1%)	0.60
Functioning forms	51/72 (70.8%)	328/500 (65.6%)	0.46
PPGL localization			0.43
Abdominal PGL	23/95 (24.3%)	149/646 (23.1%)	
Mediastinal PGL	0/95 (0.0%)	2/646 (0.3%)	
HNPGL	0/95 (0.0%)	3/646 (0.4%)	
PCC	2/95 (2.1%)	35/646 (5.4%)	
Abdominal PGL + PCC	4/95 (4.2%)	54/646 (8.4%)	
Mediastinal PGL + HNPGL	6/95 (6.3%)	50/646 (7.7%)	
Abdominal PGL + HNPGL	0/95 (0.0%)	5/646 (0.8%)	
PCC + HNPGL	60/95 (63.1%)	348/646 (53.9%)	
Positive family history of cancer	36/89 (40.4%)	228/638 (35.7%)	0.45
Risk factors			
Smoke	21/83 (25.3%)	138/589 (23.4%)	0.81
Alcohol	4/86 (4.7%)	28/592 (4.7%)	1.00
Exposure to toxic substances	3/77 (3.9%)	26/548 (4.7%)	0.97
Germ-line mutation	10/56 (17.9%)	156/459 (34.0%)	**0.01**
Genetic test			
Wild type	46/56 (82.1%)	303/459 (66.0%)	0.18
*SDHA*	0/56 (0.0%)	4/459 (0.9%)	1.00
*SDHB*	1/56 (1.7%)	44/459 (9.6%)	0.08
*SDHC*	0/56 (0.0%)	7/459 (1.5%)	0.73
*SDHD*	5/56 (8.3%)	81/459 (17.2%)	0.12
*SDHAF2*	0/56 (0.0%)	1/459 (0.2%)	1.00
*MAX*	2/56 (3.6%)	10/459 (2.2%)	0.86
*TMEM127*	2/56 (3.5%)	9/459 (2.0%)	0.79
Cluster 1	6/54 (11.1%)	137/459 (29.8%)	**0.006**
Cluster 2	4/54 (7.4%)	19/459 (4.1%)	0.45
Years between PPGL and second malignant tumor, median	6 (IQR: 2–14)		
Death	7/95 (7.4%)	19/646 (2.9%)	0.06
Death for PPGL	3/95 (3.2%)	8/646 (1.2%)	0.32
Follow up months, median	36 (IQR: 12–108)	48 (IQR: 15–108)	0.47

PPGL = pheochromocytoma and paraganglioma; IQR = interquartile range; PGL = paraganglioma; HNPGL = Head and neck paraganglioma; PCC = pheochromocytoma. Statistically significant *p* are indicated in bold.

**Table 4:** Univariable analysis for incident second malignant tumor.

	HR	95% CI		*p* Value
Males vs. females	0.79	0.45	1.39	0.42
Age category				
50–59 years vs. <50 years	2.27	1.13	4.53	**0.021**
>60 years vs. <50 years	2.22	1.05	4.69	**0.036**
Metastatic PPGLs (yes vs. no)	0.20	0.03	1.49	0.12
Functioning PPGLs (no vs. yes)	0.80	0.38	1.66	0.54
Parasympathetic vs. sympathetic lesions	0.89	0.50	1.60	0.71
Family history of cancer (yes vs. no)	1.80	1.03	3.14	**0.04**
Germ-line mutation vs. wild type	0.27	0.11	0.63	**0.003**
Cluster 1 (positive vs. negative)	0.31	0.13	0.73	**0.008**
Cluster 2 (positive vs. negative)	0.82	0.24	2.74	0.75
Risk factors (yes vs. no)				
Smoke	1.18	0.58	2.40	0.64
Alcohol	2.46	0.75	8.06	0.14
Exposure to toxic substances	0.57	0.01	4.11	0.67

PPGL = pheochromocytoma and paraganglioma. Statistically significant *p* are indicated in bold.

**Table 5:** Multivariable analysis for the risk of developing a malignant tumor excluding patients with a second tumor developed before or simultaneously with the chromaffin tumor (A) and limited to the patients with genetic evaluation (B).

	A (*n*. 741)				B (*n*. 515)			
	HR	95% CI		*p* Value	HR	95% CI		*p* Value
Age category								
50–59 years vs. <50 years	2.50	1.15	5.44	**0.021**	1.71	0.72	4.07	0.23
>60 years vs. <40 years	3.46	1.67	7.15	**<0.001**	1.46	0.54	3.94	0.46
Males vs. females	1.23	0.63	2.41	0.54	1.18	0.53	2.60	0.69
Smoker vs. non-smoker	2.10	0.82	5.39	0.12	1.64	0.57	4.72	0.36
Genetic test (positive vs. negative)					0.25	0.10	0.63	**0.003**

Statistically significant *p* are indicated in bold.

## Data Availability

The data presented in this study are available in this article.

## Supplementary Materials

The following are available online at https://www.mdpi.com/article/10.3390/cancers13225831/s1, Available in FigShare (10.6084/m9.figshare.14737494). Table S1: Association between non-chromaffin tumors and PCC/PGL and/or mutations in one of the PCC/PGL susceptibly genes doi.10.6084/m9.figshare.14737494.

## Appendix A

AIRTUM Working Group—Collaborators.

Bisceglia, I.; Candela, G.; Carozzi, G.; Cavallo, R., Celesia, M.V.; Cirilli, C.; Citarella, A.; Contiero, P.; Cuccaro, F.; Dal Maso, L.; Fusco, M.; Galasso, R.; Giuliani, O.; Mangone, L.; Marani, E.; Maule, M.; Mazzoleni, G.; Melcarne, A.; Michiara, M.; Musolino, A.; Paderni, F.; Palma, F.; Piffer, S.; Pompili, M.; Quarta, F.; Ravaioli, A.; Rizzello, R.; Rugge, M.; Sacerdote, C.; Sciacchitano, C.G.; Serraino, D.; Sferrazza, A.; Sutera Sardo, A.; Tagliabue, G.; Tumino, R.; Valenti Clemente, S.; Vincenzi, R.; Vitale, M.F.; Vitarelli, S.; Vittadello, F.

**NAME**	**SURNAME**	**TUMOR REGISTRY**
Guido	Mazzoleni	South Tyrol Tumour Registry, Italy
Fabio	Vittadello	South Tyrol Tumour Registry, Italy
Rosario	Tumino	Cancer Registry, Provincial Health Authority (ASP) Ragusa, Italy
Ausilia	Sferrazza	Cancer Registry, Provincial Health Authority (ASP) Ragusa, Italy
Marco	Pompili	Cancer Registry Marche, Italy
Susanna	Vitarelli	Cancer Registry Marche, Italy
Francesco	Cuccaro	Cancer Registry of Puglia, Italy
Giuseppa	Candela	Cancer Registry Trapani-Agrigento ASP Trapani, Italy
Roberto	Rizzello	Trento Province Cancer Registry, Trento, Italy
Silvano	Piffer	Trento Province Cancer Registry, Trento, Italy
Maria	Michiara	Cancer Registry of Parma, Parma, Italy
Antonino	Musolino	Cancer Registry of Parma, Parma, Italy
Milena	Maule	Unit of Cancer Epidemiology, Turin, Italy
Carlotta	Sacerdote	Unit of Cancer Epidemiology, Turin, Italy
Alessandra	Ravaioli	Cancer Registry Romagna, Italy
Orietta	Giuliani	Cancer Registry Romagna, Italy
Maria Vittoria	Celesia	Cancer Registry Liguria, Italy
Enza	Marani	Cancer Registry Liguria, Italy
Diego	Serraino	Oncology referral Center Aviano, Italy
Luigino	Dal Maso	Oncology referral Center Aviano, Italy
Fernando	Palma	Cancer Registry Foggia, Section Cancer Registry Puglia, Italy
Mario	Fusco	Napoli 3 South Cancer Registry, Italy
Maria Francesca	Vitale	Napoli 3 South Cancer Registry, Italy
Giuliano	Carozzi	Modena Cancer Registry, Italy
Claudia	Cirilii	Modena Cancer Registry, Italy
Giovanna	Tagliabue	Lombardy Cancer Registry, Italy
Paolo	Contiero	Lombardy Cancer Registry, Italy
Santa	Valenti Clemente	Reggio Calabria Tumour Registry, Italy
Romina	Vincenzi	Reggio Calabria Tumour Registry, Italy
Rocco	Galasso	Regional Cancer Registry Basilicata, Italy
Fabrizio	Quarta	Lecce Tumour Registry, Italy
Anna	Melcarne	Lecce Tumour Registry, Italy
Rossella	Cavallo	Salerno Tumour Registry, Italy
Lucia	Mangone	Reggio-Emilia Tumour Registry, Italy
Isabella	Bisceglia	Reggio-Emilia Tumour Registry, Italy
Carlo Giacomo	Sciacchitano	CT-ME-EN Tumour Registry, Italy
Fiorella	Paderni	CT-ME-EN Tumour Registry, Italy
Annarita	Citarella	Benevento Tumour Registry, Italy
Antonella	Sutera Sardo	Catanzaro Tumour Registry, Italy
Massimo	Rugge	Veneto Tumour Registry, Italy

## Appendix B

List of 12 referral centers in Italy involved in the study.

1. Istituto Oncologico Veneto IRCCS, Padova;
2. Dipartimento di Scienze Biomediche Sperimentali e Cliniche, AOU Careggi, Firenze;
3. Centro Specialistico Ipertensioni Secondarie, Dipartimento di Medicina Interna e Specialità Mediche, Università di Roma “Sapienza”, Policlinico Umberto I, Roma;
4. Endocrinologia, Diabetologia e Metabolismo, Dipartimento di Scienze Mediche, Università di Torino, Città della Salute e della Scienza, Torino e Endocrinologia Oncologica, Dipartimento di Scienze Mediche, Università di Torino, Città della Salute e della Scienza, Torino;
5. Medicina Interna ed Endocrinologia, Dipartimento di Scienze Cliniche e Biologiche, Università di Torino, AOU San Luigi, Orbassano Torino;
6. Unità di Endocrinologia e Malattie Metaboliche Fondazione IRCCS Cà Granda Ospedale Maggiore Policlinico, Milano;
7. Dipartimento di medicina Clinica e Chirurgia, Divisione di Endocrinologia Università Federico II Napoli;
8. Endocrinologia AO S. Croce e Carle, Cuneo;
9. Unità di Endocrinologia Istituto Nazionale Tumori Regina Elena, Roma;
10. Medicina Interna e Ipertensione, Dipartimento di Scienze Mediche, Università di Torino, Città della Salute e della Scienza, Torino;
11. Endocrinologia, Dipartimento di Medicina Clinica e Molecolare, Università Roma “Sapienza”, Ospedale Sant’Andrea, Roma;
12. Endocrinologia, AO Ordine Mauriziano, Torino
