# Supplementary material for: A Multicenter Epidemiological Study on Second Malignancy in Non-Syndromic Pheochromocytoma/Paraganglioma Patients in Italy
Source: Cancers (Basel). 2021 Nov 20;13(22):5831. doi: 10.3390/cancers13225831 (PMC8616182; doi:10.3390/cancers13225831)
Supplement: Supplementary file 1 [file cancers-13-05831-s001.zip › cancers-1465083-supplementary/cancers-1465083-supplementary Table S1.pdf]

	Study population	Cases	SDH negative immunohistochemistry	Loss of heterozygosity (LOH)
Morris MR et al., 2004[1]	51 RCC and 4 oncocytomas	29 RCC	0/0	0/29
Vanharanta S et al., 2004[2]	352 Pheo/PGL patients	3 RCC in *SDHB* patients (2/3 with PGL)<br>60 sporadic RCC<br>35 kidney cancers with clear cell histology	0/0	3 *SDHB* mutations/3<br><br>0/60<br>0/35
Benn DE et al., 2006[3]	116 patients (83 affected and 33 clinically unaffected) from 62 families with PCC/PGL syndromes and *SDHB* or *SDHD* mutations	1 GIST	1 SDHB-/1	0/0
McWhinney SR et al., 2007[4]	9 *SDHx* GIST	9 GIST	0/0	5 *SDHB* mutations, 2 *SDHC* mutations, 1 *SDHD* mutation/8
Solis DC et al., 2009[5]	41 *SDHB* patients	1 papillary thyroid carcinoma<br>1 uterine leiomyoma<br>1 HNPGL + hybrid renal tumor<br>1 HNPGL + papillary bladder carcinoma<br>1 HNPGL + basocellular carcinoma precordial<br>1 lung cancer	0/0	1 *SDHB* mutation in hybrid renal tumor /1 RCC + 0/1 PTC
Gill AJ et al., 2010[6]	121 GIST (104 sporadic, 5 Carney Triad, 3 NF1, 2 cKIT mutated, 1 multifocal GIST, 1 pediatric, 5 in young people)	121 GIST	10 SDHB- (3 sporadic, 5 Carney Triad, 1 pediatric, 1 multifocal)/121	0/0
Housley SL et al., 2010[7]	1 *SDHB* patient affected by RCC	1 RCC	0/0	1 *SDHB* mutation/1
Gaal J et al., 2011[8]	57 GIST<br>(4 Carney Stratakis, 6 Carney Triad, 5 cKIT or PDGFRA mutated, 42	1 medullary thyroid carcinoma in patient with sporadic GIST<br><br>57 GIST	0/1<br><br><br><br>11 SDHB-/57	1 *RET* mutation/1<br><br><br>0/0

	sporadic), 1 medullary thyroid carcinoma		(4/4 Carney Stratakis, 6/6 Carney Triad, 0/5 cKIT or PDGFRA mu, 1/42 sporadic)	
Gill AJ et al., 2011[9]*	4 *SDHB* patients affected by 5 RCC	5 RCC	4 SDHB-/4	0/0
Gill AJ et al., 2011[10]	3 RCC in SDHB patients	3 RCC	3 SDHB-/3	0/0
Janeway KA et al., 2011[11]	34 GIST (30 wt patients, 1 *SDHC*, 3 *SDHB*)	34 GIST	2 SDHB- (2 *SDHB* patients)/3 (2 *SDHB* patients, 1 *SDHC* patient)	0/0
Miettinen M et al., 2011[12]	1134 GIST	1134 GIST	66 SDHB-/1134	0/0
Alvarenga CA et al., 2012[13]	1 patient affected by chromophobe RCC + PGL	1 chromophobe RCC	0/0	0/1
Doyle LA et al. 2012[14]	264 GIST (179 cKIT mutated,<br>32 PDGFRA mutated,<br>53 wt)	264 GIST	22 SDHB-/264	0/0
Italiano A et al. 2012[15]	28 GIST in young adults	28 GIST	4 SDHB- (3/4 SDHB-/SDHA-)/7	3 *SDHA* mutations/28
Malinoc A et al., 2012[16]	35 *SDHC* patients + 1 proband's mother	2 RCC<br>(1 clear cell cancer, a papillary carcinoma,	1 SDHB- (clear cell cancer)/2	2 *SDHC* mutations/2
Xekouki P et al., 2012[17]	1 PA (GH) in *SDHD* patient	1 PA	0/0	1 *SDHD* mutation/1
Xekouki P et al., 2012[18]	1 PA (macroGH) + PGLs + PCCs in *SDHD* patient	1 PA	1 SDHB-/1	1 *SDHD* mutation/1
Belinsky MG et al., 2013[19]	24 GIST<br>(12 wt, 12 KIT/PDGFRA/BRAF mutated)	24 GIST	11 SDHB- (3/11 wt GIST in Carney Triad)/24	4 *SDHA* mutations/6
Dwight T et al., 2013[20]	Colonic polyps + HNPGL<br><br>1 (NS) in *SDHA* patient	Colonic polyps<br><br><br>1 PA	0/1<br><br><br>1 SDHB- and A-/1	0/0<br><br><br>0/1
Dwight T et al., 2013[21]	10 patients with SDH-deficient GIST	10 GIST	10 SDHB- (3/10 SDHB- and A-)/10	3 *SDHA* mutations/10
Gill AJ et al., 2013[22]	GIST + leiomyosarcoma + unclassified renal tumor + PTC in *SDHC* patient	1 leiomyosarcoma<br>1 PTC<br>1 GIST<br>1 RCC	1 SDHB-/3 (1 PTC, 1 GIST, 1 RCC)	0/0
Kim S et al., 2013[23]	721 breast cancers	721 breast cancers	41 SDHA-/319<br>24 SDHB-/245	0/0

Miettinen M et al., 2013[24]	944 GIST	944 GIST	127 SDHB- (36/127 SDHB-/A-)/944	6 *SDHA* mutations/7
Oudijk L et al. 2013[25]	33 GIST	33 GIST	16 SDHB- (4/16 SDHB-/A-, one associated with metastatic medullary thyroid carcinoma)/33	4 *SDHA* mutations/4
Papathomas TG et al., 2014[26]	348 tumors	5 RCC<br>2 PA	4 SDHB-/4<br>1 SDHB-/2	4/4<br>1/2
Wagner AJ et al. 2013[27]	33 GIST	33 GIST	33 SDHB- (9/33 SDHB- and A-)/33	9 *SDHA* mutations/9
Choi J et al., 2014[28]	206 filloid breast tumors	206 filloid breast tumors	36 SDHB- (5/36 SDHB- and A-)/206	0/0
Fleming S et al., 2014[29]	3 RCC in *SDHB* patients (one previously described[§])	2 RCC	2 SDHB-/2	0/0
Gill AJ et al., 2014[30]	36 RCC[‡] in 27 *SDHx* patients	36 RCC	36 SDHB-/36	0/0
Gill AJ et al., 2014[31]	309 PA	309 PA	1 SDHB-/A- (macro PRL)/309	1 *SDHA* mutation/1
Miettinen M et al., 2014[32]	2258 epithelial tumors	1547 other tumors<br><br>711 RCC	3 SDHB- (1 prostate adenocarcinoma, 1 stomach adenocarcinoma, 1 testis seminoma)/1547<br><br>4 SDHB-<br>(1 clear cell carcinoma<br>1 papillary carcinoma<br>2 unclassified carcinomas)/711	0/0<br><br>0/0
Paik JY et al., 2014[33]	1 RCC in *SDHB* patient	1 RCC	1 SDHB-/1	0/0
Pantaleo MA et al., 2014[34]	358 GIST (34 KIT/PDGFRA wt)	34 wt KIT/PDGFRA GIST	6 SDHB– (4 SDHB- and A-)/20	6 *SDHA* mutations, 1 *SDHB* mutation, 1 *SDHC* mutation/34
Renella R et al., 2014[35]	1 *SDHB* patient affected by lymphoma + PGL	1 lymphoma	1 markedly reduced immunoreactivity/1	0/0
Tirumani SH et al., 2014[36]*	34 *SDHx* GIST	34 GIST	34 SDHB-/34	0/0
Cornejo KM et al. 2015[37]	420 renal epithelial tumors	420 renal epithelial tumors	0/420	0/0
Dénes J et al., 2015[38]	39 patients (19 sporadic, 20 familial)	23 PA: 12 PRL, 6 GH, 5 NFPA (5 *SDHB*, 1 *SDHC*, 1	1 SDHB-/7	3 SDHB mutations/5

		SDHA, 1 *SDHAF2*, 15 no mut)		
Jiang Q et al. 2015[39]	1 GIST + chromophobe renal carcinoma + kidney cist in a *SDHA* patient	1 GIST<br><br>1 chromophobe renal carcinoma	1 SDHB-/1<br><br>1 SDHB-/1	1 *SDHA* mutation/1<br><br>1 *SDHA* mutation/1
Ni Y et al., 2015[40]	802 patients affected by differentiated thyroid carcinomas or breast carcinomas + data from TGCA thyroid cancer (THCA) dataset (476 patients)	754 patients	0/0	5 (3 *SDHB* and 2 *SDHD* mutations)/37 apparently sporadic epithelial thyroid carcinomas (7 follicular, 4 follicular variant of papillary histology, 26 PTC)
Niemeijer ND et al., 2015[41]	26 *SDHx* patients affected by 35 non paragangliomic tumors	4 *SDHx* patients with 5 different tumors<br><br>2 GIST<br><br>3 RCC<br><br>2 PA	2 SDHB- (ganglioneuroma and neuroendocrine pancreas tumor)/5<br><br>2 SDHB-/2<br><br>3 SDHB-/3<br><br>2 SDHB- (1/2 SDHB-/A-)/2	1 *SDHD* mutation (neuroendocrine pancreas tumor)/2<br><br>1 *SDHD*, 1 *SDHB* mutation/2<br>3 *SDHB* mutations /3<br><br>1 *SDHD* mutation (+ 1 somatic mutation)/2
O'Toole SM et al., 2015[42]	72 literature cases	60 new cases [#]	0/0	2 *SDHB* mutations/ number of cases not known
Ozluk Y et al., 2015[43]	1 metastatic RCC	1 RCC	1 SDHB- and A-/1	1 *SDHA* mutation/1
Pantaleo MA et al., 2015[44]	69 metastatic GIST	11 KIT/PDGFRA wild-type GIST	6 SDHB-/11	6 *SDHA* mutations/6
Williamson SR et al., 2015[45]	37 RCC with histology in line with SDH	37 RCC	11 SDHB- (1 SDHB- and A-/11)/37	5 *SDHB* mutations/6
Yakirevich E et al. 2015[46]	1 RCC	1 RCC	1 SDHB- and A-/1	1 *SDHA* mutation/1
Falzarano SM et al., 2016[47]	7 RCC	7 RCC	0/7	0/0
Kim E et al., 2017[48]	11 RCC, 10 GIST	11 RCC, 10 GIST	11 SDHB-/11 RCC<br>10 SDHB-/10 GIST	0/0
Mason EF et al., 2016[49][$]	705 GIST	76 GIST	76 SDHB- (28/76 SDHB-/A-)/76	0/0
Belinsky MG et al., 2017[50]	A GIST	1 GIST	1 SDHB-/1	1 *SDHB* mutation/1
Caliò A et al., 2017[51]	4 RCC (3/4 *SDHB* patients)	4 RCC	4 SDHB-/4	0/0

Carter CS et al., 2017[52]	96 cutaneous leiomyomas from 87 patients (12/96 from 7 patients with documented HLRCC)	96 cutaneous leiomyomas from 87 patients	9 FH- (7/9 from HLRCC patients) + 2 equivocal<br>1 equivocal SDHB /96	0/0
Casey R et al., 2017[53]	12 patients affected by PCC/PGL/HNPGL + RCC (1/12 + breast carcinoma)	1 oncocytoma, 11 RCC	0/0	1 *MAX* mutation/1
De Sousa SMC et al., 2017[54]	44 patients affected by PA	2 parathyroid tumors<br>1 NFPA<br>1 microPRL<br>1 PRL<br>1 GH + pituitary gangliocytoma<br>1 PA	0/2 parathyroid tumors<br>0/1 PA	0/0
Else T et al., 2017[55]	4 *SDHx* cortisol secreting ACC	4 *SDHx* cortisol secreting ACC	0/3	1 possible LOH/4
Elston MS et al., 2017[56]	1 GIST in *SDHB* patient	1 GIST in *SDHB* patient	1 SDHB- and SDHA+/1	1 *SDHB* mutation/1
Gupta S et al., 2017[57]	14 RCC + PGL (3 affected by VHL)	11 RCC	0/11	0/0
Gupta S et al., 2017[58]	1 composite PGL-ganglioneuroma, 7 composite PCC-ganglioneuromas	1 composite PGL-ganglioneuroma, 7 composite PCC-ganglioneuromas	0/8	0/0
Iwashita H et al., 2017[59]	1 RCC	1 RCC	1 SDHB-/1	1 *SDHB* mutation/1
Liu W et al., 2017[60]	355 GIST	355 GIST	12 SDHB- /355	0/0
Schaefer IM et al., 2017[61]	76 GIST	76 GIST	14 SDHB-/22	17 MAX mutations/76
Tufton N et al., 2017[62]	1 pituitary carcinoma (NF) + HNPGL in *SDHB* patient	1 PA	0/0	2 somatic *SDHA* inactivating mutations/1
Kumar R et al., 2018[63]	1 papillary RCC	1 RCC	1 SDHB-/1	0/0
Li Y et al., 2018[64]	33 unclassified RCC	33 RCC	8 SDHB- and A+, 4 FH-/33	0/0
Maher M et al., 2018[65]	1 PA (macroPRL)	1 PA	0/1	Partial retention of the normal allele at the mutation site of *SDHB* gene/1
McEvoy CR et al., 2018[66]	1 RCC	1 RCC	1 SDHB- and A -/1	1 *SDHA* likely pathogenic mutation/1
Michalova K et al., 2018[67]	10 papillary RCC	10 papillary RCC	0/10	0/0
Williamson SR et al., 2018[68]	12 RCC† + 16 clear cell papillary renal cell	12 RCC† + 16 clear cell papillary renal cell	28 SDHB-/34<br>Equivocal in 1 unclassified	0/0

	carcinoma tumors + 6 unclassified tumors	carcinoma tumors + 6 unclassified tumors	tumor	
Brcic I et al., 2019[69]	1 GIST	1 GIST	1 SHDB- and A- /1	1 *SDHA* mutation/1
Casey RT et al., 2019[70]	15 GIST	15 GIST	10 SDHB-/15	0/0
Chatzopoulos K et al., 2019[71]	6 pulmonary chondroma, 33 pulmonary hamartoma	6 pulmonary chondroma, 33 pulmonary hamartoma	0/33<br>5/6 (4 affected by Carney triad)	0/0
Gupta S et al., 2019[72]	1009 RCC (400 papillary, 203 chromophobe, 87 chromophobe, 273 oncocytomas, 46 unclassified)	1009 RCC	3 SDHB- (initially classified ad oncocytomas)/1009	0/0
Kalfusova A et al., 2019[73]	261 GIST	44 GIST (without KIT, PDGFRA, and BRAF mutations)	0/0	1 deletion of the whole *SDHB* gene, deletion of exons 2–4 of the *SDHC* gene, and deletion of exons 2–4 of the *SDHD* gene and 1 deletion of *SDHAF2* exons 1-4 and deletion of *SDHB* exons 1-4/4
Kennedy JM et al., 2019[74]	23 biopsies and 37 resections from 30 patients affected by HRCCS	1 RCC in *SDHD* patient	1 SDHB-/1	0/0
Malik F et al., 2019[75]	1 GIST with 2 different morphological population: one well differentiated and one dedifferentiated	1 GIST	1 SDHB-/1	1 *SDHB* mutation/1
Nicolas E et al., 2019[76]	1 RCC + PTC	1 RCC	1 SDHB -/1	0/1
Ricci R et al., 2019[77]	48 GIST<br>(5 in 3 *NF1* patients were excluded)	43 GIST	9 SDHB- /34	7 *SDHA* mutations, 1 *SDHB* mutation, 1 *SDHC* mutation/9
Roh TH et al., 2019[78]	90 CNS tumors	17 glioblastomas<br>7 astrocytomas<br>9 oligodendroglyomas<br>9 ependymomas<br>7 central neurocytomas	3 SDHB–(hemangioblastomas)/90	0/0

	35 hemangioblastomas	3 schwannomas<br>10 meningiomas<br>6 hemangiopericytomas<br>3 hemangioblastomas<br>5 craniopharyngiomas<br>12 pituitary adenomas<br><br>35 hemangioblastomas	3 SDHB-/35	0/0
Saavedra A et al., 2019[79]	1 macroPA (GH) + metastatic PGL in *SDHB* patient	1 PA	1 heterogeneus staining pattern for SDHB/1	0/1
Shi SS et al., 2019[80]	366 GIST	26 GIST	26 SDHB-/26	10 missense mutations (6 *SDHD*, 3 *SDHB*, 1 *SDHC*)/26
Stanley K et al., 2019[81]	1 *SDHC* patient affected by GIST + renal and neck cysts	1 GIST	1 SDHB-/1	0/0
Urgate-Camara M et al., 2019[82]	2 RCC in a *SDHB* patient	2 RCC	0 (1 doubt to review)/2	0/0
De Filpo G et al., 2020[83]	1 *SDHB* patient	1 mediastinal germ cell tumor	0/1	0/1
Gokozan HN et al., 2020[84]	1 GIST	1 GIST	1 SDHB-/1	0/0
Heilig CE et al., 2020[85]	1 metastatic high-grade spindle cell sarcoma not otherwise specified	1 metastatic high-grade spindle cell sarcoma not otherwise specified in *SDHB* patient	0/0	1 *SDHB* mutation/1
Wilczek Y et al., 2020[86]	1 RCC	1 RCC	1 SDHB-/1	0/0

Supplementary Table S1: association between non-chromaffin tumors and PCC/PGL and/or mutations in one of the PCC/PGL susceptibly genes.

ACC adrenocortical carcinoma, CNS central nervous system, FH fumarate hydratase, GH growth hormone, HLRCC Hereditary Leiomyomatosis and Renal Cell Carcinoma, HNPGL head and neck paraganglioma, NFPA non-functioning pituitary adenoma, PGL sympathetic paraganglioma, PCC pheochromocytoma, PRL prolactin, PTC papillary thyroid carcinoma, RCC renal cell carcinoma, VUS variant of uncertain significance.

*included RCC reported in Gill AJ et al., 2011[9] and Housley SL et al., 2010[7]

§ Housley SL et al., 2010[7]

‡ 21/36 previously unreported cases

13 PA +PCC + known mutations, 21 PA + PCC + suspect clinical picture, 26 PA + PCC

$ partial temporal overlap between these two studies (Tirumani SH et al., 2014[36] and Mason EF et al., 2016[49]): some patients may have been included in both studies.

† 9/12 RCC previously reported in Williamson SR et al., 2015[45]

REFERENCES

1. Morris, M.R.; Maina, E.; Morgan, N.V.; Gentle, D.; Astuti, D.; Moch, H.; Kishida, T.; Yao, M.; Schraml, P.; Richards, F.M.; et al. Molecular genetic analysis of FIH-1, FH, and SDHB candidate tumour suppressor genes in renal cell carcinoma. *J Clin Pathol* **2004**, *57*, 706-711, doi:10.1136/jcp.2003.011767.
2. Vanharanta, S.; Buchta, M.; McWhinney, S.R.; Virta, S.K.; Peçzkowska, M.; Morrison, C.D.; Lehtonen, R.; Januszewicz, A.; Järvinen, H.; Juhola, M.; et al. Early-onset renal cell carcinoma as a novel extraparaganglial component of SDHB-associated heritable paraganglioma. *Am J Hum Genet* **2004**, *74*, 153-159, doi:10.1086/381054.
3. Benn, D.E.; Gimenez-Roqueplo, A.P.; Reilly, J.R.; Bertherat, J.; Burgess, J.; Byth, K.; Croxson, M.; Dahia, P.L.; Elston, M.; Gimm, O.; et al. Clinical presentation and penetrance of pheochromocytoma/paraganglioma syndromes. *J Clin Endocrinol Metab* **2006**, *91*, 827-836, doi:10.1210/jc.2005-1862.
4. McWhinney, S.R.; Pasini, B.; Stratakis, C.A.; International Carney, T.; Carney-Stratakis Syndrome, C. Familial gastrointestinal stromal tumors and germ-line mutations. *N Engl J Med* **2007**, *357*, 1054-1056, doi:10.1056/NEJMc071191.
5. Solis, D.C.; Burnichon, N.; Timmers, H.J.; Raygada, M.J.; Kozupa, A.; Merino, M.J.; Makey, D.; Adams, K.T.; Venisse, A.; Gimenez-Roqueplo, A.P.; et al. Penetrance and clinical consequences of a gross SDHB deletion in a large family. *Clin Genet* **2009**, *75*, 354-363, doi:10.1111/j.1399-0004.2009.01157.x.
6. Gill, A.J.; Chou, A.; Vilain, R.; Clarkson, A.; Lui, M.; Jin, R.; Tobias, V.; Samra, J.; Goldstein, D.; Smith, C.; et al. Immunohistochemistry for SDHB divides gastrointestinal stromal tumors (GISTs) into 2 distinct types. *Am J Surg Pathol* **2010**, *34*, 636-644, doi:10.1097/PAS.0b013e3181d6150d.
7. Housley, S.L.; Lindsay, R.S.; Young, B.; McConachie, M.; Mechan, D.; Baty, D.; Christie, L.; Rahilly, M.; Qureshi, K.; Fleming, S. Renal carcinoma with giant mitochondria associated with germ-line mutation and somatic loss of the succinate dehydrogenase B gene. *Histopathology* **2010**, *56*, 405-408, doi:10.1111/j.1365-2559.2010.03482.x.
8. Gaal, J.; Stratakis, C.A.; Carney, J.A.; Ball, E.R.; Korpershoek, E.; Lodish, M.B.; Levy, I.; Xekouki, P.; van Nederveen, F.H.; den Bakker, M.A.; et al. SDHB immunohistochemistry: a useful tool in the diagnosis of Carney-Stratakis and Carney triad gastrointestinal stromal tumors. *Mod Pathol* **2011**, *24*, 147-151, doi:10.1038/modpathol.2010.185.
9. Gill, A.J.; Pachter, N.S.; Chou, A.; Young, B.; Clarkson, A.; Tucker, K.M.; Winship, I.M.; Earls, P.; Benn, D.E.; Robinson, B.G.; et al. Renal tumors associated with germline SDHB mutation show distinctive morphology. *Am J Surg Pathol* **2011**, *35*, 1578-1585, doi:10.1097/PAS.0b013e318227e7f4.
10. Gill, A.J.; Pachter, N.S.; Clarkson, A.; Tucker, K.M.; Winship, I.M.; Benn, D.E.; Robinson, B.G.; Clifton-Bligh, R.J. Renal tumors and hereditary pheochromocytoma-paraganglioma syndrome type 4. *N Engl J Med* **2011**, *364*, 885-886, doi:10.1056/NEJMc1012357.
11. Janeway, K.A.; Kim, S.Y.; Lodish, M.; Nose, V.; Rustin, P.; Gaal, J.; Dahia, P.L.; Liegl, B.; Ball, E.R.; Raygada, M.; et al. Defects in succinate dehydrogenase in gastrointestinal stromal tumors lacking KIT and PDGFRA mutations. *Proc Natl Acad Sci U S A* **2011**, *108*, 314-318, doi:10.1073/pnas.1009199108.
12. Miettinen, M.; Wang, Z.F.; Sarlomo-Rikala, M.; Osuch, C.; Rutkowski, P.; Lasota, J. Succinate dehydrogenase-deficient GISTs: a clinicopathologic, immunohistochemical, and molecular genetic study of 66 gastric GISTs with predilection to young age. *Am J Surg Pathol* **2011**, *35*, 1712-1721, doi:10.1097/PAS.0b013e3182260752.
13. Alvarenga, C.A.; Lopes, J.M.; Vinagre, J.; Paravidino, P.I.; Alvarenga, M.; Prando, A.; Castilho, L.N.; Soares, P.; Billis, A. Paraganglioma of seminal vesicle and chromophobe renal cell carcinoma: a case report and literature review. *Sao Paulo Med J* **2012**, *130*, 57-60, doi:10.1590/s1516-31802012000100010.
14. Doyle, L.A.; Nelson, D.; Heinrich, M.C.; Corless, C.L.; Hornick, J.L. Loss of succinate dehydrogenase subunit B (SDHB) expression is limited to a distinctive subset of gastric wild-type gastrointestinal stromal tumours: a comprehensive genotype-phenotype correlation study. *Histopathology* **2012**, *61*, 801-809, doi:10.1111/j.1365-2559.2012.04300.x.
15. Italiano, A.; Chen, C.L.; Sung, Y.S.; Singer, S.; DeMatteo, R.P.; LaQuaglia, M.P.; Besmer, P.; Socci, N.; Antonescu, C.R. SDHA loss of function mutations in a subset of young adult wild-type gastrointestinal stromal tumors. *BMC Cancer* **2012**, *12*, 408, doi:10.1186/1471-2407-12-408.
16. Malinoc, A.; Sullivan, M.; Wiech, T.; Schmid, K.W.; Jilg, C.; Straeter, J.; Deger, S.; Hoffmann, M.M.; Bosse, A.; Rasp, G.; et al. Biallelic inactivation of the SDHC gene in renal carcinoma associated with paraganglioma syndrome type 3. *Endocr Relat Cancer* **2012**, *19*, 283-290, doi:10.1530/ERC-11-0324.
17. Xekouki, P.; Stratakis, C.A. Succinate dehydrogenase (SDHx) mutations in pituitary tumors: could this be a new role for mitochondrial complex II and/or Krebs cycle defects? *Endocr Relat Cancer* **2012**, *19*, C33-40, doi:10.1530/ERC-12-0118.
18. Xekouki, P.; Pacak, K.; Almeida, M.; Wassif, C.A.; Rustin, P.; Nesterova, M.; de la Luz Sierra, M.; Matro, J.; Ball, E.; Azevedo, M.; et al. Succinate dehydrogenase (SDH) D subunit (SDHD) inactivation in a growth-hormone-producing pituitary tumor: a new association for SDH? *J Clin Endocrinol Metab* **2012**, *97*, E357-366, doi:10.1210/jc.2011-1179.

19. Belinsky, M.G.; Rink, L.; Flieder, D.B.; Jahromi, M.S.; Schiffman, J.D.; Godwin, A.K.; Mehren, M. Overexpression of insulin-like growth factor 1 receptor and frequent mutational inactivation of SDHA in wild-type SDHB-negative gastrointestinal stromal tumors. *Genes Chromosomes Cancer* **2013**, *52*, 214-224, doi:10.1002/gcc.22023.
20. Dwight, T.; Mann, K.; Benn, D.E.; Robinson, B.G.; McKelvie, P.; Gill, A.J.; Winship, I.; Clifton-Bligh, R.J. Familial SDHA mutation associated with pituitary adenoma and pheochromocytoma/paraganglioma. *J Clin Endocrinol Metab* **2013**, *98*, E1103-1108, doi:10.1210/jc.2013-1400.
21. Dwight, T.; Benn, D.E.; Clarkson, A.; Vilain, R.; Lipton, L.; Robinson, B.G.; Clifton-Bligh, R.J.; Gill, A.J. Loss of SDHA expression identifies SDHA mutations in succinate dehydrogenase-deficient gastrointestinal stromal tumors. *Am J Surg Pathol* **2013**, *37*, 226-233, doi:10.1097/PAS.0b013e3182671155.
22. Gill, A.J.; Lipton, L.; Taylor, J.; Benn, D.E.; Richardson, A.L.; Frydenberg, M.; Shapiro, J.; Clifton-Bligh, R.J.; Chow, C.W.; Bogwitz, M. Germline SDHC mutation presenting as recurrent SDH deficient GIST and renal carcinoma. *Pathology* **2013**, *45*, 689-691, doi:10.1097/PAT.0000000000000018.
23. Kim, S.; Kim, D.H.; Jung, W.H.; Koo, J.S. Succinate dehydrogenase expression in breast cancer. *Springerplus* **2013**, *2*, 299, doi:10.1186/2193-1801-2-299.
24. Miettinen, M.; Killian, J.K.; Wang, Z.F.; Lasota, J.; Lau, C.; Jones, L.; Walker, R.; Pineda, M.; Zhu, Y.J.; Kim, S.Y.; et al. Immunohistochemical loss of succinate dehydrogenase subunit A (SDHA) in gastrointestinal stromal tumors (GISTs) signals SDHA germline mutation. *Am J Surg Pathol* **2013**, *37*, 234-240, doi:10.1097/PAS.0b013e3182671178.
25. Oudijk, L.; Gaal, J.; Korpershoek, E.; van Nederveen, F.H.; Kelly, L.; Schiavon, G.; Verweij, J.; Mathijssen, R.H.; den Bakker, M.A.; Oldenburg, R.A.; et al. SDHA mutations in adult and pediatric wild-type gastrointestinal stromal tumors. *Mod Pathol* **2013**, *26*, 456-463, doi:10.1038/modpathol.2012.186.
26. Papathomas, T.G.; Gaal, J.; Corssmit, E.P.; Oudijk, L.; Korpershoek, E.; Heimdal, K.; Bayley, J.P.; Morreau, H.; van Dooren, M.; Papaspyrou, K.; et al. Non-pheochromocytoma (PCC)/paraganglioma (PGL) tumors in patients with succinate dehydrogenase-related PCC-PGL syndromes: a clinicopathological and molecular analysis. *Eur J Endocrinol* **2014**, *170*, 1-12, doi:10.1530/EJE-13-0623.
27. Wagner, A.J.; Remillard, S.P.; Zhang, Y.X.; Doyle, L.A.; George, S.; Hornick, J.L. Loss of expression of SDHA predicts SDHA mutations in gastrointestinal stromal tumors. *Mod Pathol* **2013**, *26*, 289-294, doi:10.1038/modpathol.2012.153.
28. Choi, J.; Kim, D.H.; Jung, W.; Koo, J.S. The expression of succinate dehydrogenase in breast phyllodes tumor. *Histol Histopathol* **2014**, *29*, 1343-1354, doi:10.14670/HH-29.1343.
29. Fleming, S.; Mayer, N.J.; Vlatkovic, L.J.; McLean, J.; McConachie, M.; Baty, D. Signalling pathways in succinate dehydrogenase B-associated renal carcinoma. *Histopathology* **2014**, *64*, 477-483, doi:10.1111/his.12250.
30. Gill, A.J.; Hes, O.; Papathomas, T.; Šedivcová, M.; Tan, P.H.; Agaimy, A.; Andresen, P.A.; Kedziora, A.; Clarkson, A.; Toon, C.W.; et al. Succinate dehydrogenase (SDH)-deficient renal carcinoma: a morphologically distinct entity: a clinicopathologic series of 36 tumors from 27 patients. *Am J Surg Pathol* **2014**, *38*, 1588-1602, doi:10.1097/PAS.0000000000000292.
31. Gill, A.J.; Toon, C.W.; Clarkson, A.; Sioson, L.; Chou, A.; Winship, I.; Robinson, B.G.; Benn, D.E.; Clifton-Bligh, R.J.; Dwight, T. Succinate dehydrogenase deficiency is rare in pituitary adenomas. *Am J Surg Pathol* **2014**, *38*, 560-566, doi:10.1097/PAS.0000000000000149.
32. Miettinen, M.; Sarlomo-Rikala, M.; McCue, P.; Czapiewski, P.; Langfort, R.; Waloszczyk, P.; Wazny, K.; Biernat, W.; Lasota, J.; Wang, Z. Mapping of succinate dehydrogenase losses in 2258 epithelial neoplasms. *Appl Immunohistochem Mol Morphol* **2014**, *22*, 31-36, doi:10.1097/PAI.0b013e31828bfdd3.
33. Paik, J.Y.; Toon, C.W.; Benn, D.E.; High, H.; Hasovitz, C.; Pavlakis, N.; Clifton-Bligh, R.J.; Gill, A.J. Renal carcinoma associated with succinate dehydrogenase B mutation: a new and unique subtype of renal carcinoma. *J Clin Oncol* **2014**, *32*, e10-13, doi:10.1200/JCO.2012.47.2647.
34. Pantaleo, M.A.; Astolfi, A.; Urbini, M.; Nannini, M.; Paterini, P.; Indio, V.; Saponara, M.; Formica, S.; Ceccarelli, C.; Casadio, R.; et al. Analysis of all subunits, SDHA, SDHB, SDHC, SDHD, of the succinate dehydrogenase complex in KIT/PDGFRA wild-type GIST. *Eur J Hum Genet* **2014**, *22*, 32-39, doi:10.1038/ejhg.2013.80.
35. Renella, R.; Carnevale, J.; Schneider, K.A.; Hornick, J.L.; Rana, H.Q.; Janeway, K.A. Exploring the association of succinate dehydrogenase complex mutations with lymphoid malignancies. *Fam Cancer* **2014**, *13*, 507-511, doi:10.1007/s10689-014-9725-4.
36. Tirumani, S.H.; Tirumani, H.; Jagannathan, J.P.; Shinagare, A.B.; Hornick, J.L.; George, S.; Wagner, A.J.; Ramaiya, N.H. MDCT features of succinate dehydrogenase (SDH)-deficient gastrointestinal stromal tumours. *Br J Radiol* **2014**, *87*, 20140476, doi:10.1259/bjr.20140476.
37. Cornejo, K.M.; Lu, M.; Yang, P.; Wu, S.; Cai, C.; Zhong, W.D.; Olumi, A.; Young, R.H.; Wu, C.L. Succinate dehydrogenase B: a new prognostic biomarker in clear cell renal cell carcinoma. *Hum Pathol* **2015**, *46*, 820-826, doi:10.1016/j.humpath.2015.02.013.
38. Denes, J.; Swords, F.; Rattenberry, E.; Stals, K.; Owens, M.; Cranston, T.; Xekouki, P.; Moran, L.; Kumar, A.; Wassif, C.; et al. Heterogeneous genetic background of the association of pheochromocytoma/paraganglioma and pituitary adenoma: results from a large patient cohort. *J Clin Endocrinol Metab* **2015**, *100*, E531-541, doi:10.1210/jc.2014-3399.
39. Jiang, Q.; Zhang, Y.; Zhou, Y.H.; Hou, Y.Y.; Wang, J.Y.; Li, J.L.; Li, M.; Tong, H.X.; Lu, W.Q. A novel germline mutation in SDHA identified in a rare case of gastrointestinal stromal tumor complicated with renal cell carcinoma. *Int J Clin Exp Pathol* **2015**, *8*, 12188-12197.
40. Ni, Y.; Seballos, S.; Ganapathi, S.; Gurin, D.; Fletcher, B.; Ngeow, J.; Nagy, R.; Kloos, R.T.; Ringel, M.D.; LaFramboise, T.; et al. Germline and somatic SDHx alterations in apparently sporadic differentiated thyroid cancer. *Endocr Relat Cancer* **2015**, *22*, 121-130, doi:10.1530/ERC-14-0537.

41. Niemeijer, N.D.; Papathomas, T.G.; Korpershoek, E.; de Krijger, R.R.; Oudijk, L.; Morreau, H.; Bayley, J.P.; Hes, F.J.; Jansen, J.C.; Dinjens, W.N.; et al. Succinate Dehydrogenase (SDH)-Deficient Pancreatic Neuroendocrine Tumor Expands the SDH-Related Tumor Spectrum. *J Clin Endocrinol Metab* **2015**, *100*, E1386-1393, doi:10.1210/jc.2015-2689.
42. O'Toole, S.M.; Dénes, J.; Robledo, M.; Stratakis, C.A.; Korbonits, M. 15 YEARS OF PARAGANGLIOMA: The association of pituitary adenomas and phaeochromocytomas or paragangliomas. *Endocr Relat Cancer* **2015**, *22*, T105-122, doi:10.1530/ERC-15-0241.
43. Ozluk, Y.; Taheri, D.; Matoso, A.; Sanli, O.; Berker, N.K.; Yakirevich, E.; Balasubramanian, S.; Ross, J.S.; Ali, S.M.; Netto, G.J. Renal carcinoma associated with a novel succinate dehydrogenase A mutation: a case report and review of literature of a rare subtype of renal carcinoma. *Hum Pathol* **2015**, *46*, 1951-1955, doi:10.1016/j.humpath.2015.07.027.
44. Pantaleo, M.A.; Lolli, C.; Nannini, M.; Astolfi, A.; Indio, V.; Saponara, M.; Urbini, M.; La Rovere, S.; Gill, A.; Goldstein, D.; et al. Good survival outcome of metastatic SDH-deficient gastrointestinal stromal tumors harboring SDHA mutations. *Genet Med* **2015**, *17*, 391-395, doi:10.1038/gim.2014.115.
45. Williamson, S.R.; Eble, J.N.; Amin, M.B.; Gupta, N.S.; Smith, S.C.; Sholl, L.M.; Montironi, R.; Hirsch, M.S.; Hornick, J.L. Succinate dehydrogenase-deficient renal cell carcinoma: detailed characterization of 11 tumors defining a unique subtype of renal cell carcinoma. *Mod Pathol* **2015**, *28*, 80-94, doi:10.1038/modpathol.2014.86.
46. Yakirevich, E.; Ali, S.M.; Mega, A.; McMahon, C.; Brodsky, A.S.; Ross, J.S.; Allen, J.; Elvin, J.A.; Safran, H.; Resnick, M.B. A Novel SDHA-deficient Renal Cell Carcinoma Revealed by Comprehensive Genomic Profiling. *Am J Surg Pathol* **2015**, *39*, 858-863, doi:10.1097/PAS.0000000000000403.
47. Falzarano, S.M.; McKenney, J.K.; Montironi, R.; Eble, J.N.; Osunkoya, A.O.; Guo, J.; Zhou, S.; Xiao, H.; Dhanasekaran, S.M.; Shukla, S.; et al. Renal Cell Carcinoma Occurring in Patients With Prior Neuroblastoma: A Heterogenous Group of Neoplasms. *Am J Surg Pathol* **2016**, *40*, 989-997, doi:10.1097/PAS.0000000000000632.
48. Kim, E.; Wright, M.J.; Sioson, L.; Novos, T.; Gill, A.J.; Benn, D.E.; White, C.; Dwight, T.; Clifton-Bligh, R.J. Utility of the succinate: Fumarate ratio for assessing SDH dysfunction in different tumor types. *Mol Genet Metab Rep* **2017**, *10*, 45-49, doi:10.1016/j.ymgmr.2016.12.006.
49. Mason, E.F.; Hornick, J.L. Conventional Risk Stratification Fails to Predict Progression of Succinate Dehydrogenase-deficient Gastrointestinal Stromal Tumors: A Clinicopathologic Study of 76 Cases. *Am J Surg Pathol* **2016**, *40*, 1616-1621, doi:10.1097/PAS.0000000000000685.
50. Belinsky, M.G.; Cai, K.Q.; Zhou, Y.; Luo, B.; Pei, J.; Rink, L.; von Mehren, M. Succinate dehydrogenase deficiency in a PDGFRA mutated GIST. *BMC Cancer* **2017**, *17*, 512, doi:10.1186/s12885-017-3499-7.
51. Calio, A.; Grignon, D.J.; Stohr, B.A.; Williamson, S.R.; Eble, J.N.; Cheng, L. Renal cell carcinoma with TFE3 translocation and succinate dehydrogenase B mutation. *Mod Pathol* **2017**, *30*, 407-415, doi:10.1038/modpathol.2016.200.
52. Carter, C.S.; Skala, S.L.; Chinnaiyan, A.M.; McHugh, J.B.; Siddiqui, J.; Cao, X.; Dhanasekaran, S.M.; Fullen, D.R.; Lagstein, A.; Chan, M.P.; et al. Immunohistochemical Characterization of Fumarate Hydratase (FH) and Succinate Dehydrogenase (SDH) in Cutaneous Leiomyomas for Detection of Familial Cancer Syndromes. *Am J Surg Pathol* **2017**, *41*, 801-809, doi:10.1097/PAS.0000000000000840.
53. Casey, R.T.; Warren, A.Y.; Martin, J.E.; Challis, B.G.; Rattenberry, E.; Whitworth, J.; Andrews, K.A.; Roberts, T.; Clark, G.R.; West, H.; et al. Clinical and Molecular Features of Renal and Pheochromocytoma/Paraganglioma Tumor Association Syndrome (RAPTAS): Case Series and Literature Review. *J Clin Endocrinol Metab* **2017**, *102*, 4013-4022, doi:10.1210/jc.2017-00562.
54. De Sousa, S.M.C.; McCabe, M.J.; Wu, K.; Roscioli, T.; Gayevskiy, V.; Brook, K.; Rawlings, L.; Scott, H.S.; Thompson, T.J.; Earls, P.; et al. Germline variants in familial pituitary tumour syndrome genes are common in young patients and families with additional endocrine tumours. *Eur J Endocrinol* **2017**, *176*, 635-644, doi:10.1530/EJE-16-0944.
55. Else, T.; Lerario, A.M.; Everett, J.; Haymon, L.; Wham, D.; Mullane, M.; Wilson, T.L.; Rainville, I.; Rana, H.; Worth, A.J.; et al. Adrenocortical carcinoma and succinate dehydrogenase gene mutations: an observational case series. *Eur J Endocrinol* **2017**, *177*, 439-444, doi:10.1530/EJE-17-0358.
56. Elston, M.S.; Sehgal, S.; Dray, M.; Phillips, E.; Conaglen, J.V.; Clifton-Bligh, R.J.; Gill, A.J. A Duodenal SDH-Deficient Gastrointestinal Stromal Tumor in a Patient With a Germline SDHB Mutation. *J Clin Endocrinol Metab* **2017**, *102*, 1447-1450, doi:10.1210/jc.2017-00165.
57. Gupta, S.; Zhang, J.; Milosevic, D.; Mills, J.R.; Grebe, S.K.; Smith, S.C.; Erickson, L.A. Primary Renal Paragangliomas and Renal Neoplasia Associated with Pheochromocytoma/Paraganglioma: Analysis of von Hippel-Lindau (VHL), Succinate Dehydrogenase (SDHX) and Transmembrane Protein 127 (TMEM127). *Endocr Pathol* **2017**, *28*, 253-268, doi:10.1007/s12022-017-9489-0.
58. Gupta, S.; Zhang, J.; Erickson, L.A. Composite Pheochromocytoma/Paraganglioma-Ganglioneuroma: A Clinicopathologic Study of Eight Cases with Analysis of Succinate Dehydrogenase. *Endocr Pathol* **2017**, *28*, 269-275, doi:10.1007/s12022-017-9494-3.
59. Iwashita, H.; Okudela, K.; Matsumura, M.; Yamanaka, S.; Sawazumi, T.; Enaka, M.; Udaka, N.; Miyake, A.; Hibiya, T.; Miyake, N.; et al. Succinate dehydrogenase B-deficient renal cell carcinoma: A case report with novel germline mutation. *Pathol Int* **2017**, *67*, 585-589, doi:10.1111/pin.12587.
60. Liu, W.; Zeng, X.; Wu, X.; He, J.; Gao, J.; Shuai, X.; Wang, G.; Zhang, P.; Tao, K. Clinicopathologic study of succinate-dehydrogenase-deficient gastrointestinal stromal tumors: A single-institutional experience in China. *Medicine (Baltimore)* **2017**, *96*, e7668, doi:10.1097/MD.0000000000007668.
61. Schaefer, I.M.; Wang, Y.; Liang, C.W.; Bahri, N.; Quattrone, A.; Doyle, L.; Marino-Enriquez, A.; Lauria, A.; Zhu, M.; Debiec-Rychter, M.; et al. MAX inactivation is an early event in GIST development that regulates p16 and cell proliferation. *Nat Commun* **2017**, *8*, 14674, doi:10.1038/ncomms14674.

62. Tufton, N.; Roncaroli, F.; Hadjidemetriou, I.; Dang, M.N.; Denes, J.; Guasti, L.; Thom, M.; Powell, M.; Baldeweg, S.E.; Fersht, N.; et al. Pituitary Carcinoma in a Patient with an SDHB Mutation. *Endocr Pathol* **2017**, *28*, 320-325, doi:10.1007/s12022-017-9474-7.
63. Chidambaram, V.; Tun, N.L.; Haque, W.Z.; Majella, M.G.; Sivakumar, R.K.; Kumar, A.; Hsu, A.T.; Ishak, I.A.; Nur, A.A.; Ayeh, S.K.; et al. Factors associated with disease severity and mortality among patients with COVID-19: A systematic review and meta-analysis. *PLoS One* **2020**, *15*, e0241541, doi:10.1371/journal.pone.0241541.
64. Li, Y.; Reuter, V.E.; Matoso, A.; Netto, G.J.; Epstein, J.I.; Argani, P. Re-evaluation of 33 'unclassified' eosinophilic renal cell carcinomas in young patients. *Histopathology* **2018**, *72*, 588-600, doi:10.1111/his.13395.
65. Maher, M.; Roncaroli, F.; Mendoza, N.; Meeran, K.; Canham, N.; Kosicka-Slawinska, M.; Bernhard, B.; Collier, D.; Drummond, J.; Skordilis, K.; et al. A patient with a germline SDHB mutation presenting with an isolated pituitary macroprolactinoma. *Endocrinol Diabetes Metab Case Rep* **2018**, *2018*, doi:10.1530/EDM-18-0078.
66. McEvoy, C.R.; Koe, L.; Choong, D.Y.; Leong, H.S.; Xu, H.; Karikios, D.; Plew, J.D.; Prall, O.W.; Fellowes, A.P.; Fox, S.B. SDH-deficient renal cell carcinoma associated with biallelic mutation in succinate dehydrogenase A: comprehensive genetic profiling and its relation to therapy response. *NPJ Precis Oncol* **2018**, *2*, 9, doi:10.1038/s41698-018-0053-2.
67. Michalova, K.; Steiner, P.; Alaghehbandan, R.; Trpkov, K.; Martinek, P.; Grossmann, P.; Montiel, D.P.; Sperga, M.; Straka, L.; Prochazkova, K.; et al. Papillary renal cell carcinoma with cytologic and molecular genetic features overlapping with renal oncocytoma: Analysis of 10 cases. *Ann Diagn Pathol* **2018**, *35*, 1-6, doi:10.1016/j.anndiagpath.2018.01.010.
68. Williamson, S.R.; Hornick, J.L.; Eble, J.N.; Gupta, N.S.; Rogers, C.G.; True, L.; Grignon, D.J.; Cheng, L. Renal cell carcinoma with angioleiomyoma-like stroma and clear cell papillary renal cell carcinoma: exploring SDHB protein immunohistochemistry and the relationship to tuberous sclerosis complex. *Hum Pathol* **2018**, *75*, 10-15, doi:10.1016/j.humpath.2017.11.013.
69. Brcic, I.; Kashofer, K.; Skone, D.; Liegl-Atzwanger, B. KIT mutation in a naive succinate dehydrogenase-deficient gastric GIST. *Genes Chromosomes Cancer* **2019**, *58*, 798-803, doi:10.1002/gcc.22768.
70. Casey, R.T.; Ten Hoopen, R.; Ochoa, E.; Challis, B.G.; Whitworth, J.; Smith, P.S.; Martin, J.E.; Clark, G.R.; Rodger, F.; Maranian, M.; et al. SDHC epi-mutation testing in gastrointestinal stromal tumours and related tumours in clinical practice. *Sci Rep* **2019**, *9*, 10244, doi:10.1038/s41598-019-46124-9.
71. Chatzopoulos, K.; Fritchie, K.J.; Aubry, M.C.; Carney, J.A.; Folpe, A.L.; Boland, J.M. Loss of succinate dehydrogenase B immunohistochemical expression distinguishes pulmonary chondromas from hamartomas. *Histopathology* **2019**, *75*, 825-832, doi:10.1111/his.13945.
72. Gupta, S.; Swanson, A.A.; Chen, Y.B.; Lopez, T.; Milosevic, D.; Kipp, B.R.; Leibovich, B.C.; Thompson, R.H.; Herrera-Hernandez, L.; Cheville, J.C.; et al. Incidence of succinate dehydrogenase and fumarate hydratase-deficient renal cell carcinoma based on immunohistochemical screening with SDHA/SDHB and FH/2SC. *Hum Pathol* **2019**, *91*, 114-122, doi:10.1016/j.humpath.2019.07.004.
73. Kalfusova, A.; Linke, Z.; Kalinova, M.; Krskova, L.; Hilska, I.; Szabova, J.; Vicha, A.; Kodet, R. Gastrointestinal stromal tumors - Summary of mutational status of the primary/secondary KIT/PDGFRA mutations, BRAF mutations and SDH defects. *Pathol Res Pract* **2019**, *215*, 152708, doi:10.1016/j.prp.2019.152708.
74. Kennedy, J.M.; Wang, X.; Plouffe, K.R.; Dhanasekaran, S.M.; Hafez, K.; Palapattu, G.S.; Else, T.; Weizer, A.Z.; Morgan, T.M.; Spratt, D.E.; et al. Clinical and morphologic review of 60 hereditary renal tumors from 30 hereditary renal cell carcinoma syndrome patients: lessons from a contemporary single institution series. *Med Oncol* **2019**, *36*, 74, doi:10.1007/s12032-019-1297-6.
75. Malik, F.; Santiago, T.; Bahrami, A.; Davis, E.; McCarville, B.; Newman, S.; Azzato, E.M.; Davidoff, A.M.; Brennan, R.; Ellison, D.W.; et al. Dedifferentiation in SDH-Deficient Gastrointestinal Stromal Tumor: A Report With Histologic, Immunophenotypic, and Molecular Characterization. *Pediatr Dev Pathol* **2019**, *22*, 492-498, doi:10.1177/1093526619846222.
76. Nicolas, E.; Demidova, E.V.; Iqbal, W.; Serebriiskii, I.G.; Vlasenkova, R.; Ghatalia, P.; Zhou, Y.; Rainey, K.; Forman, A.F.; Dunbrack, R.L., Jr.; et al. Interaction of germline variants in a family with a history of early-onset clear cell renal cell carcinoma. *Mol Genet Genomic Med* **2019**, *7*, e556, doi:10.1002/mgg3.556.
77. Ricci, R.; Martini, M.; Ravegnini, G.; Cenci, T.; Milione, M.; Lanza, P.; Pierconti, F.; Santini, D.; Angelini, S.; Biondi, A.; et al. Preferential MGMT methylation could predispose a subset of KIT/PDGFRA-WT GISTs, including SDH-deficient ones, to respond to alkylating agents. *Clin Epigenetics* **2019**, *11*, 2, doi:10.1186/s13148-018-0594-9.
78. Roh, T.H.; Yim, H.; Roh, J.; Lee, K.B.; Park, S.H.; Jeong, S.Y.; Kim, S.H.; Kim, J.H. The loss of succinate dehydrogenase B expression is frequently identified in hemangioblastoma of the central nervous system. *Sci Rep* **2019**, *9*, 5873, doi:10.1038/s41598-019-42338-z.
79. Saavedra, A.; Lima, J.; Castro, L.; Silva, R.; Macedo, S.; Rodrigues, E.; Carvalho, D. Malignant paraganglioma and somatotropinoma in a patient with germline SDHB mutation-genetic and clinical features. *Endocrine* **2019**, *63*, 182-187, doi:10.1007/s12020-018-1726-x.
80. Shi, S.S.; Wang, Y.F.; Bao, W.; Ye, S.B.; Wu, N.; Wang, X.; Xia, Q.Y.; Li, R.; Shen, Q.; Zhou, X.J. Genetic and epigenetic alterations of SDH genes in patients with sporadic succinate dehydrogenase-deficient gastrointestinal stromal tumors. *Pathol Int* **2019**, *69*, 350-359, doi:10.1111/pin.12809.
81. Stanley, K.; Friehling, E.; Davis, A.; Ranganathan, S. Succinate Dehydrogenase-Deficient Gastrointestinal Stromal Tumor With SDHC Germline Mutation and Bilateral Renal and Neck Cysts. *Pediatr Dev Pathol* **2019**, *22*, 265-268, doi:10.1177/1093526618805354.
82. Ugarte-Camara, M.; Fernandez-Prado, R.; Lorda, I.; Rossello, G.; Gonzalez-Enguita, C.; Cannata-Ortiz, P.; Ortiz, A. Positive/retained SDHB immunostaining in renal cell carcinomas associated to germline SDHB-deficiency: case report. *Diagn Pathol* **2019**, *14*, 42, doi:10.1186/s13000-019-0812-6.

83. De Filpo, G.; Cilotti, A.; Rolli, L.; Pastorino, U.; Sonzogni, A.; Pradella, S.; Cantini, G.; Ercolino, T.; Nesi, G.; Mannelli, M.; et al. SDHx and Non-Chromaffin Tumors: A Mediastinal Germ Cell Tumor Occurring in a Young Man with Germline SDHB Mutation. *Medicina (Kaunas)* **2020**, *56*, doi:10.3390/medicina56110561.
84. Gokozan, H.N.; Bomeisl, P. Succinate dehydrogenase-deficient gastrointestinal stromal tumor of stomach diagnosed by endoscopic ultrasound-guided fine-needle biopsy: Report of a distinct subtype in cytology. *Diagn Cytopathol* **2020**, *48*, 1328-1332, doi:10.1002/dc.24591.
85. Heilig, C.E.; Horak, P.; Lipka, D.B.; Mock, A.; Uhrig, S.; Kreutzfeldt, S.; Richter, S.; Gieldon, L.; Frohlich, M.; Hutter, B.; et al. Germline SDHB-inactivating mutation in gastric spindle cell sarcoma. *Genes Chromosomes Cancer* **2020**, *59*, 601-608, doi:10.1002/gcc.22876.
86. Wilczek, Y.; Sachdeva, A.; Turner, H.; Veeratterapillay, R. SDH-deficient renal cell carcinoma: a clinicopathological analysis highlighting the role of genetic counselling. *Ann R Coll Surg Engl* **2021**, *103*, e20-e22, doi:10.1308/rcsann.2020.0196.
